# Double burden of malnutrition and its associated factors among adolescents in Debre Berhan Regiopolitan City, Ethiopia: a multinomial regression model analysis

**DOI:** 10.3389/fnut.2023.1187875

**Published:** 2023-07-18

**Authors:** Lemma Getacher, Beyene Wondafrash Ademe, Tefera Belachew

**Affiliations:** ^1^School of Public Health, Asrat Woldeyes Health Science Campus, Debre Berhan University, Debre Berhan, Ethiopia; ^2^Department of Nutrition and Dietetics, Faculty of Public Health, Institute of Health, Jimma University, Jimma, Ethiopia

**Keywords:** double burden of malnutrition, school adolescents, overnutrition, undernutrition, Ethiopia

## Abstract

**Background:**

The double burden of malnutrition (DBM), contained both undernutrition and overnutrition, is a growing public health concern that presents a significant challenge to the food and nutrition policies of developing nations such as Ethiopia. However, the prevalence and contributing factors of DBM among adolescents in the study area have not been adequately investigated by Ethiopian researchers. Therefore, this study aims to determine the prevalence of DBM and contributing factors among secondary school students in Debre Berhan City, Ethiopia.

**Methods:**

A school-based cross-sectional study was conducted among 742 adolescents aged 10–19 years from October 13, 2022, to November 14, 2022, using a multi-stage sampling method. Data were collected using the online Kobo toolbox tool. A multinomial logistic regression model was used to analyze the data. The data were cleaned and analyzed in R software 4.2.2. Adolescents who had body mass index for age Z score (BAZ) < −2 SD, > +1 SD, and > +2 from the median value were considered thin, overweight, and obese, respectively.

**Results:**

The overall prevalence of DBM was 21.5% (14.8% thinness and 6.7% overweight/obesity). In the multivariable multinomial logistic regression analysis models factors such as age [AOR = 0.79, 95% CL: (0.67, 0.93)], sex [AOR = 3.86, 95% CL: (2.35, 6.32)], school type [AOR 5.03, 95% CL: (2.30, 10.99)], minimum dietary diversity score [AOR = 2.29, 95% CL: (1.27, 4.14)], frequency of meals [AOR = 2.09, 95% CL: (1.13, 3.89)], home gardening practice [AOR = 2.31, 95% CL: (1.44, 3.67)], history of illness [AOR = 0.57, 95% CL: (0.36, 0.93)], and knowledge of nutrition [AOR = 4.96, 95% CL: (1.61, 15.33)] were the significant predictors of either thinness or overweight/obesity (DBM).

**Conclusion:**

More than one-fifth of adolescents were affected by DBM in the study area. This prevalence is higher compared with the national and regional prevalence that found to be a public health concern. Thus, interventions like double-duty interventions should consider the age, sex, school type, minimum dietary diversity score, frequency of meals, home gardening practice, history of illness, and nutritional knowledge of adolescents.

**Clinical Trial Registration:**

clinicaltrial.gov, identifier NCT05574842.

## 1. Introduction

Undernutrition and overweight or obesity coexisting in the same individuals, households, and populations at the national and international levels throughout the life course are known as the Double Burden of Malnutrition (DBM). For instance, when DBM is present at the individual level, it may manifest as obesity combined with a vitamin or mineral deficit, or as overweight in an adult who was stunted as a child. Moreover, it can be coexist as stunted mother with stunted child at the household level and underweight children and overweight adolescents in one community or city at the population level (1–3). The Food and Agriculture Organization (FAO) and World Health Organization (WHO) initially introduced the DBM concept at the International Conference on Nutrition (ICN) in 1992 (4).

In the past, undernutrition and overnutrition were seen as distinct problems affecting various groups with various risk factors. However, it is becoming more common for these two types of malnutrition to co-occur in the same groups of people, such as overweight and stunted individuals, families, and even towns (5–8). Evidence suggests that early nutrition, diet quality, biology-related factors, epigenetics, food environments, food systems, socioeconomic issues, and weak governance were the common predictors and drivers of DBM (5, 9–11). Of these, four of them are the most modifiable and fundamental causes of various forms of malnutrition. These include early nutrition, socioeconomic determinants, dietary quality, and food environment (5, 8).

For the reason that dramatically accelerated growth and development, adolescence, like that of infancy, is a critical time of life (12). According to the WHO, it is a period between 10 and 19 years that marks the transition from childhood to adulthood (13). It is also a time when higher food intake is necessary for their rapid growth, as a result of developmental changes connected to puberty and brain development, which result in needing appropriate and adequate diets (14, 15).

The DBM is one of the main sources of morbidity and mortality worldwide, notably in low- and middle-income countries (LMICs) among these populations. It threatens and bargains adolescents’ future health and puts their young lives at high risk. Globally, around 820 million people (1 in 9) and one-third of the world population (1 in 3) are hungry and overweight or obese, respectively (3, 11, 16–18). Furthermore, 117 million boys and 75 million girls aged 5–19 years were either moderately or severely underweight in 2016 worldwide. In the same year, there were 74 million more boys and 50 million more girls who were obese (19).

According to several researches done on adolescents in various nations throughout the world indicates, the global overall prevalence of DBM (both thinness and overweight/obesity) ranges from 22.5% to 55.6%. More particularly, the prevalence of thinness ranges from 10.1 to 30.5%, and overweight/obesity ranges from 8.6% to 12.4% (20–23). Likewise, in Ethiopia, the overall prevalence of DBM (both thinness and overweight/obesity) ranges from 9.7% to 48.9%. More specifically, the prevalence of thinness ranges from 4.7% to 46.2%, and overweight/obesity ranges from 2.7% to 27.2% (24–34).

The cause of DBM can be categorized as immediate causes (inadequate/excess dietary intake and diseases), underlying causes (inadequate access to food, inadequate healthcare practice, inadequate health services, and unhealthy environment), and basic causes (poor knowledge of nutrition, lack of education, poor socioeconomic status, cultural beliefs, poor agricultural practices, poverty, poor governance, and disparities in households) (3, 35–37).

The DBM has enormous consequences on adolescents’ health which may affect the life course of adolescence in different ways. The greater risk of chronic disorders such as diabetes, hypertension, and some cancers is linked with being overweight in adolescence. Moreover, adolescents are more vulnerable to food marketing, which exposes them to obesogenic foods. Poor educational accomplishment and adverse psychosocial difficulties are also the consequences of adolescent obesity and undernutrition. On the other hand, thinness in adolescents is highly associated with a higher risk of infectious diseases. Especially, childbearing age female adolescents are adversely exposed to unwanted prenatal period outcomes such as intrauterine growth retardation, maternal mortality, stillbirth, preterm birth, and delivery complications (37–39).

Although adolescence is a period of vulnerability for DBM, most countries’ food and nutrition policies and programs in many of the LMICs and some affluent countries give little focus to the effect of DBM on the well-being and health of adolescents (9). Currently, addressing these two sorts of apparently contrasting forms of malnutrition (underweight and overweight) simultaneously brings an enormous challenge for the food and nutrition policies of developing countries like Ethiopia. Consequently, there has been a paradigm shift in thinking to reduce its effect on the health of adolescents and avert the nutritional vulnerability due to increasing linear growth, bone growth, and neurodevelopmental issue (3).

As a research gap, the prevalence and potential predictors of DBM among adolescents enrolled in secondary schools in the study area have not been examined by Ethiopian researchers. Furthermore, the association between DBM and factors such as home gardening practice, knowledge of nutrition, sex, age, type of school, and the minimum dietary diversity score of adolescents were not studied through a multinomial regression model, which is another element that was not taken into consideration in past studies. Therefore, this study aimed to determine the prevalence and associated factors of DBM in Debre Berhan City, Ethiopia.

## 2. Materials and methods

### 2.1. Study area, period, and setting

This study was conducted in Debre Berhan Regiopolitan City (DBRPC), North Shoa Zone, Amhara Region, Central Ethiopia. Debre Berhan Regiopolitan city is located 130 km away from Addis Ababa (the capital city of Ethiopia) and 690 km from Bahir Dar (the capital city of the Amhara region). It was founded by Emperor Zara Yakoob and it is the capital city of the North Shoa Zone of the Amhara region. The city has coordinated with 9°41′N 39°32′E latitude and longitude, respectively. It is found at 2,840 m above sea level, which makes it the highest city of this size in Africa. The city is subclassified into five sub-cities with an estimated number of populations of 310,254 according to the 2021/2022 municipal administrative office report of the city. There are 13,595 secondary school adolescents with 5,295 males and 8, 300 females in 10 secondary schools according to the 2022 report of the city education bureau. The city has 36 kebeles (the smallest administrative unit in Ethiopia), one comprehensive specialized hospital, 5 health centers, and about 16 health posts. This study was conducted from October 13, 2022, to November 14, 2022, in a school-based setting.

### 2.2. Study design

This study used a school-based cross-sectional study design among school adolescents in the city.

### 2.3. Population

The source population of the study was all secondary school adolescents in the city during the study period. Whereas the study population was all secondary school adolescents in the selected schools of the city during the study period. Moreover, the sample population of the study was all randomly selected sections of secondary school adolescents in the selected schools.

### 2.4. Inclusion and exclusion criteria

#### 2.4.1. Inclusion criteria

The study targeted secondary school adolescents aged 10–19 years in the study area. Secondary school adolescents who have followed their teaching-learning process in the selected schools and who had no intention of leaving that school until the end of the study were included in this study.

#### 2.4.2. Exclusion criteria

Secondary school adolescents who were not able to respond to an interview and who have a physical disability including deformities such as kyphosis, scoliosis, and limb deformity which prevents standing erect for height measurement were excluded. Moreover, participants who had confirmed diseases such as diabetes mellitus, hypertension, etc., were excluded from this study to improve the precision of anthropometric measurements and dietary practice.

### 2.5. Sample size determination

The sample size was calculated using a single proportion formula. During calculation, the following assumptions were considered: 95% confidence level, 46.2% proportion of underweight based on a previous study (40), 5% non-response rate, 1.5 design effect, and 5% marginal error, to get the maximum sample size. Accordingly, the calculated sample size was 602. However, due to the nature of cluster sampling, the final sample size of the study was included 745 study participants.

### 2.6. Sampling techniques and procedures

A multi-stage cluster sampling technique was applied to select secondary school adolescents from respective schools in DPRPC which coordinated in five sub-cities and 37 kebeles. There are 10 secondary schools in the city. Of these, eight are government and two are private schools. Six secondary schools were chosen from a pool of 10 schools using a simple random sampling technique. The sample size was distributed proportionally to these chosen secondary schools using proportional allocation to the population. The number of secondary school students in each chosen school was compiled from all of these secondary school records. The final sample students in the schools were chosen from the chosen section of selected schools. A revisit was done up to three times if an eligible study participant missed the first visit. Participants in the study were deemed to have not responded if they missed three appointments.

### 2.7. Data collection methods, procedures, and measurements

#### 2.7.1. Data collection methods and procedures

An interviewer-administered, pretested, structured questionnaire was utilized to gather the necessary data for this study. It was initially set up in English and administered face-to-face to respondents in their homes. Language experts translated this questionnaire into the Amharic spoken in the study area to make the data collection procedure operate more smoothly. The study’s data were gathered by 12 health professionals. The survey asked questions were about sociodemographic factors, adolescent dietary practice, and knowledge of nutrition.

#### 2.7.2. Measurements

With their shoes off and wearing light clothing, participants were asked to weigh themselves using a calibrated portable electronic digital scale (Seca, Germany model) to the nearest 0.1 kg. Additionally, using a portable hardwood height-measuring board with a sliding head bar and traditional anthropometric methods, the height was measured to the nearest 0.1 cm. A position for the participants was on the Frankfurt Plane. As well, the vertical stand was touched at the four locations (shoulder, calf, heel, and buttocks), and their shoes were removed. Before beginning the measurement, the stadiometer was checked using a calibration rod. All anthropometric measurements were taken twice, and if there were any differences in the measurements, the average results were chosen for analysis.

Double burden of malnutrition was the primary outcome of the study. It was measured using body mass index (BMI) of adolescents. Adolescents who had BMI for age Z score (BAZ) < −2 SD from the median value were considered as thinness. On the other hand, overweight those with BAZ > +1 SD and obese adolescents with a BAZ > +2 SD from the median value of the WHO’s 2006 reference data (41, 42). The outcome variable (DBM) which has three categories (thinness, normal, and overweight/obesity) was created by the groupings of the two indicators.

Dietary diversity score of adolescents was measured using a Minimum Dietary Diversity Score (MDDS) indicator. The dietary data was evaluated foods groups taken by adolescents during the previous 24 h. Then adolescent’s dietary diversity score was classified as adequate (≥5 food groups) or not adequate (<5 food groups) from 10 food groups. During analysis, it was coded as “1” for adequate and “0” for inadequate.

Based on six questions, knowledge of adolescents about nutrition was computed using a mean score. The questions were including about the awareness of adolescents about nutrition, dietary diversity practice on taking varieties of food groups and types of varieties of food groups they take, and understanding about cause and consequences of double burden of malnutrition. During analysis, it was coded as “1” for good knowledge and “0” for poor knowledge.

### 2.8. Data quality control

Pretest, retranslation, and contextualization of the questionnaires were done. Translations of the English language version into the Amharic language version were carried out by an Amharic language speaker who has earned a Master of Arts in the Amharic language to maintain the questionnaire’s quality and reliability. Once more, the retranslation into English was completed by a person with a Master of Arts in English. A comparison was done to preserve the consistency between the two versions. Then the questionnaire was translated and tested in Amharic before being used to collect data. The translation was compared by putting the two versions side by side by other experts other than the translators. Discrepancies were solved by discussion between the translators and comparators. The questionnaire was further reformed after the pretest. The interviewers explained every aspect of the study to the subjects while collecting the data. A practical session and a real demonstration were also included.

The data collectors and supervisors participated in a two-day intensive training session that included fieldwork and role-playing exercises before the real data gathering. Using a training document created by the research team, the training was concentrated on the methods, tools, study goals, and ethical concerns.

A standardization of anthropometric measures was conducted to lower inter-observer error. To reduce random anthropometric measurement error, the relative technical error of measurement (% TEM) was calculated. To verify the accuracy of the scale between measurements, a known object weight was employed every morning. The standardization of the anthropometrists was carried out with a coefficient of variation of less than 0.03 (3%). During training and pretesting, the precision of the data collectors’ anthropometric measures was standardized with their trainer. Adolescents had their height and weight measured twice, and the results were retaken when there was a difference of more than 0.1 kg in weight or 0.1 cm in height between the two measurements.

Every morning before data collection started, the accuracy of digital weight scales was tested using known weight, and before every weight measurement, the data collectors were confident that the scale was reading exactly at zero. Teenagers in school were urged to be themselves during the interview. Teenagers in high school who were willing to participate and who had their parents or guardians sign an assent form and an informed consent form were then questioned.

### 2.9. Data management and analysis methods

The principal investigator checked all the interviewed questionnaires visually for completeness, missing, and consistency after the data collection has been completed. To collect the data, Kobo toolbox online tool was used. The data was exported to R software version 4.2.2, and cleaned for missing values and outliers before analysis. Simple descriptive statistics such as simple frequency distribution, measures of central tendency, measures of variability, and percentages were performed to describe the demographic and socioeconomic characteristics of the respondents. Then the information was presented using tables and figures.

Descriptive statistics such as frequency tables, summary measures, and measures of dispersion were used to summarize the baseline data like sociodemographic characteristics of the adolescents. For continuous data, normality was checked using different methods such as graphical (histogram, Q-Q plot) and numerical methods (mean, median, mode, skewness, kurtosis), and statistical tests (Kolmogorov Smirnov test and Shapiro–Wilk test). Using the variance inflation factor, other assumptions multi-collinearity was checked.

To examine the data, a multinomial logistic regression model was used. To determine the relationship between each independent variable and the outcome variable in the bivariable multinomial logistic regression model, a bivariable analysis with crude odds ratio (COR) and a 95% confidence interval (CI) was performed. For independent variables that passed the bivariable test, a *p*-value cutoff less than or equal to 0.25 was employed in the model to find significant associations and control confounding effects. Using this model, it was possible to determine the variables that significantly association with DBM and obtain an adjusted odds ratio (AOR) with a 95% confidence interval. The Hosmer Lemeshaw test was used to evaluate the model’s fitness (*p* > 0.999), and standard error less than 2.0 was used to assess multicollinearity. *p*-value ≤ 0.05 was used to declare the statistical significance of the study.

## 3. Results

### 3.1. Sociodemographic characteristics of respondents

In this study, from 745 total respondents, 742 responded to the survey making a response rate of 99.6%. The mean (±SD) age and family size of the respondents were 16.74 (±1.43) and 5.19 (±1.58), respectively. Of the respondents, 31.8% were grade 9th students, 70.1% were from government schools, 69.7% were urban residents, 53.3% were female, and 94.5% were Orthodox Christian religion followers (Table 1).

**Table 1:** The sociodemographic characteristics of the respondents in the study area.

Variables	Categories	Frequency (%)
Grade level of students	Grade 9th	236 (31.8)
	Grade 10th	212 (28.6)
	Grade 11th	149 (20.1)
	Grade 12th	145 (19.5)
Type of school	Government	520 (70.1)
	Private	222 (29.9)
Age	Mean (±SD)	16.74 (±1.43)
Family size	Mean (±SD)	5.19 (±1.58)
Residence	Urban	517 (69.7)
	Rural	225 (30.3)
Sex	Male	354 (47.7)
	Female	388 (53.3)
Religion	Orthodox Tewahido	700 (94.3)
	Protestant	27 (3.6)
	Muslim	14 (1.9)
	Others	1 (0.1)
Ethnicity	Amhara	738 (99.5)
	Oromo	1 (0.1)
	Others	3 (0.4)
Mother education	Unable to read and write	226 (30.5)
	Read and write	48 (6.5)
	Primary education	163 (22.0)
	Secondary education	140 (18.9)
	More than secondary education	165 (22.2)
Mother occupation	Housewife	465 (62.7)
	Merchant	66 (8.9)
	Daily laborer	3 (0.4)
	Government employee	145 (19.5)
	Self-employed	48 (6.5)
	Others	15 (2.0)
Father education	Unable to read and write	209 (28.2)
	Read and write	162 (21.8)
	Primary education	86 (11.6)
	Secondary education	148 (19.9)
	More than secondary education	137 (18.5)
Father occupation	Farmer	280 (37.7)
	Merchant	92 (12.4)
	Daily laborer	6 (0.8)
	Government employee	171 (23.0)
	Self-employed	90 (12.1)
	Others	103 (3.9)

### 3.2. Dietary and related-practice of adolescents

Adolescents who had inadequate dietary diversity score were exposed to 12.1% and 6.1% for thinness and overweight/obesity (overnutrition), respectively. Similarly, adolescents who had poor knowledge of nutrition were exposed to 12.7% and 6.2% for thinness and overweight/obesity (overnutrition), respectively (Table 2).

**Table 2:** The crosstab distribution between the DBM and dietary and related-practice of adolescents in Debre Berhan City, Ethiopia (*n* = 742).

Variables	Categories	Double burden of malnutrition			
		Thinness (%)	Normal (%)	Overnutrition (%)	Total (%)
**Dietary diversity score**					
	Adequate	20 (2.7)	208 (28.0)	5 (0.6)	233 (31.4)
	Inadequate	90 (12.1)	374 (50.4)	45 (6.1)	509 (68.6)
	Total	110 (14.8)	582 (78.4)	50 (6.7)	742 (100)
**Frequency of meals per day**					
	<3 meals/day	24 (3.2)	56 (7.5)	22 (3.0)	102 (13.7)
	≥3 meals/day	86 (11.6)	526 (70.9)	28 (3.8)	640 (86.3)
	Total	110 (14.8)	582 (78.4)	50 (6.7)	742 (100)
**Home garden practice**					
	Yes	51 (6.9)	413 (55.7)	16 (2.2)	480 (64.7)
	No	59 (8.0)	169 (22.8)	34 (4.6)	262 (35.3)
	Total	110 (14.8)	582 (78.4)	50 (6.7)	742 (100)
**History of illness**					
	Yes	75 (10.1)	321 (43.3)	35 (4.7)	431 (58.1)
	No	35 (4.7)	261 (35.2)	15 (2.0)	311 (41.9)
	Total	110 (14.8)	582 (78.4)	50 (6.7)	742 (100)
**Knowledge of nutrition**					
	Good	16 (2.2)	213 (28.7)	4 (0.5)	233 (31.4)
	Poor	94 (12.7)	369 (49.7)	46 (6.2)	509 (68.6)
	Total	110 (14.8)	582 (78.4)	50 (6.7)	742 (100)

### 3.3. Prevalence of double burden of malnutrition

**Figure 1:**
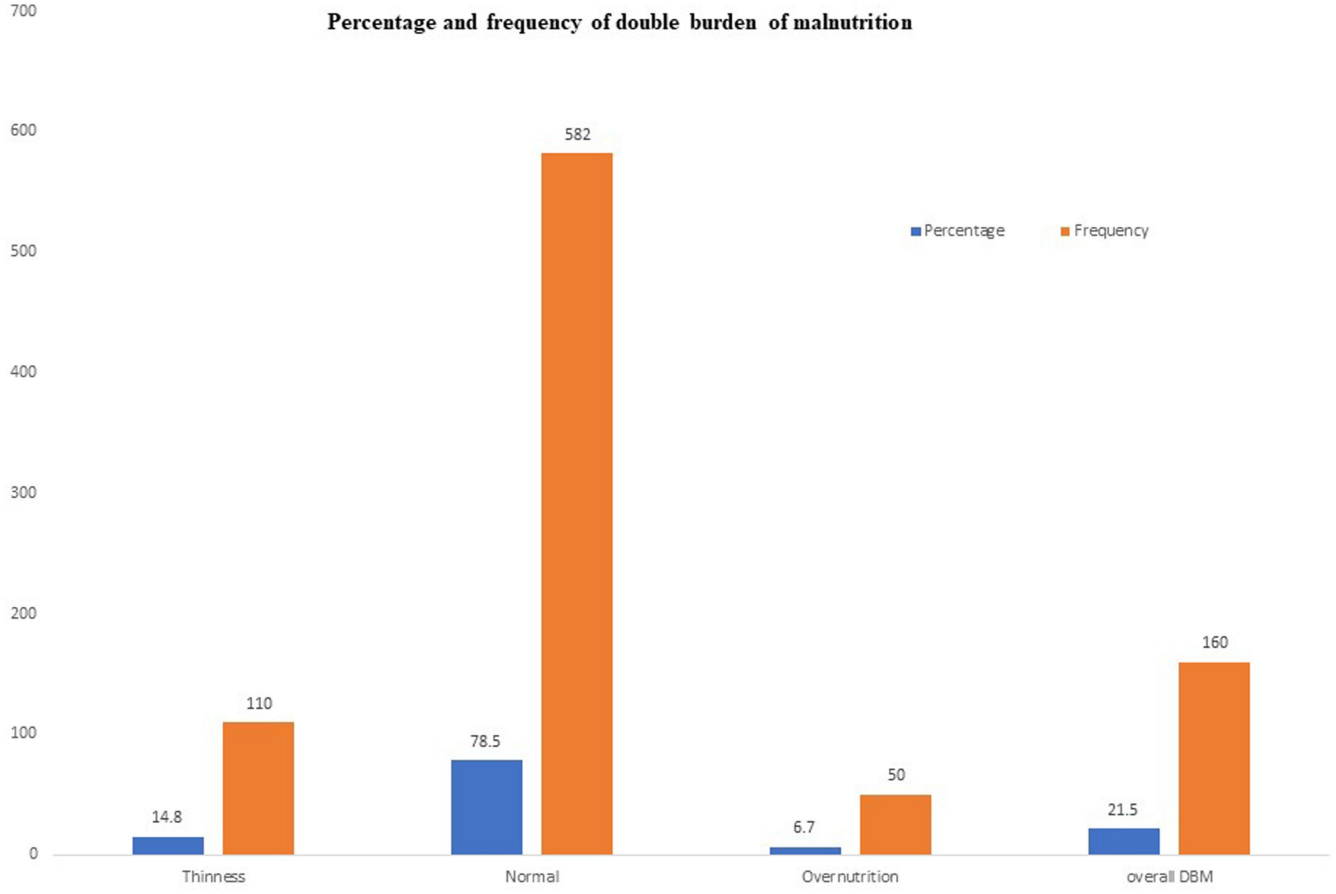
Prevalence of DBM (thinness and overweight/obesity) among school adolescents in Debre Berhan City, Ethiopia.

The summarized prevalence of DBM (both thinness and overweight/obesity) among school adolescents was 21.5%. More specifically, the prevalence of thinness among school adolescents in the study area was 14.8% [95% CL: 12.3, 17.6%] and overweight/obesity was 6.7% [95% CL: 5.0, 8.8%], respectively (Figure 1).

The mean (± SD) of the overall height and weight of the participants were 160.1 ± 7.8 cm and 49.9 (± 8.1 kg), respectively. The mean body mass index (BMI) for the age by Z-score of the participants was −0.72 (± 0.1).

**Figure 2:**
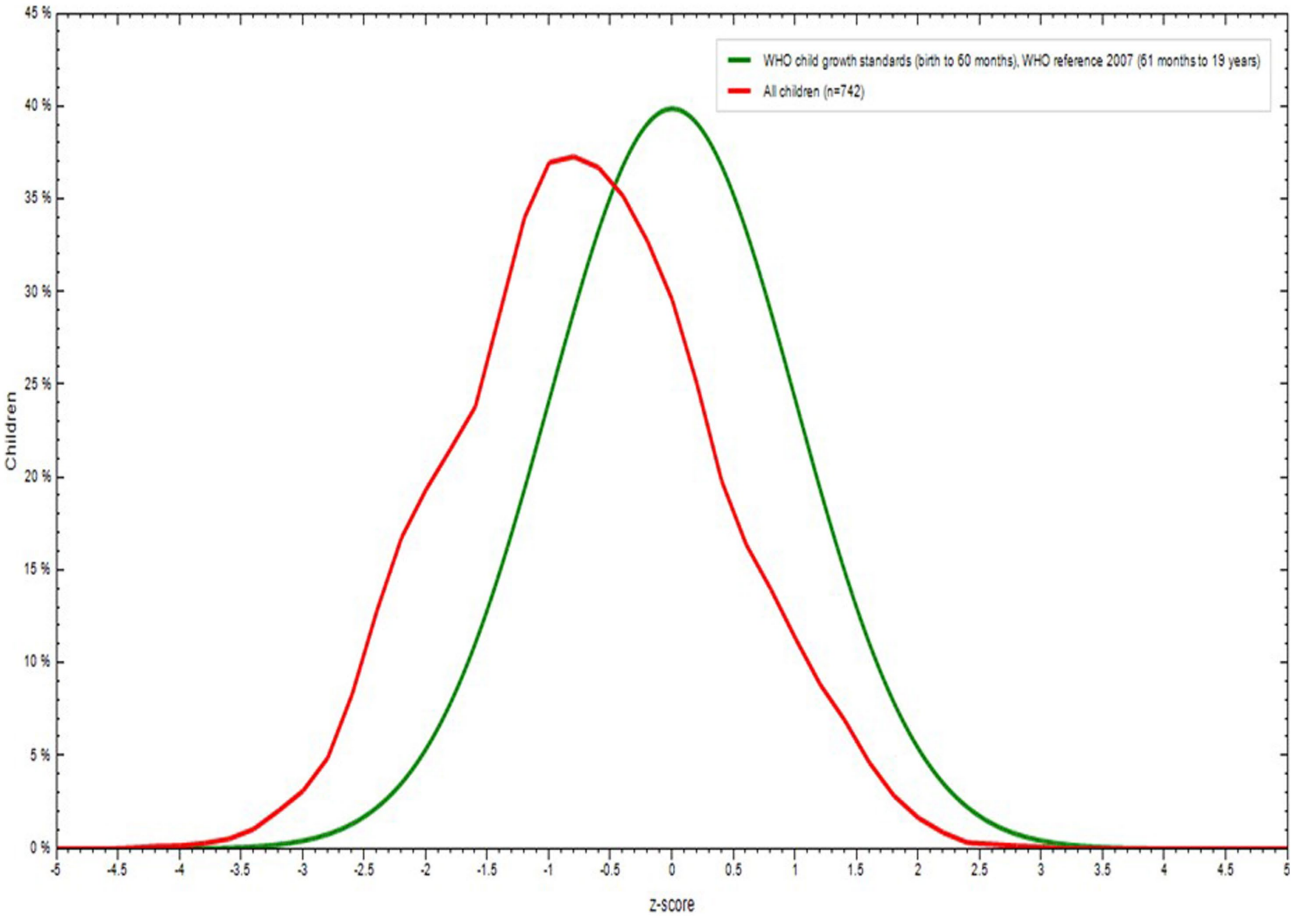
Body mass index (BMI) for Age Z score (BAZ) among school adolescents in Debre Berhan City, Ethiopia.

**Figure 3:**
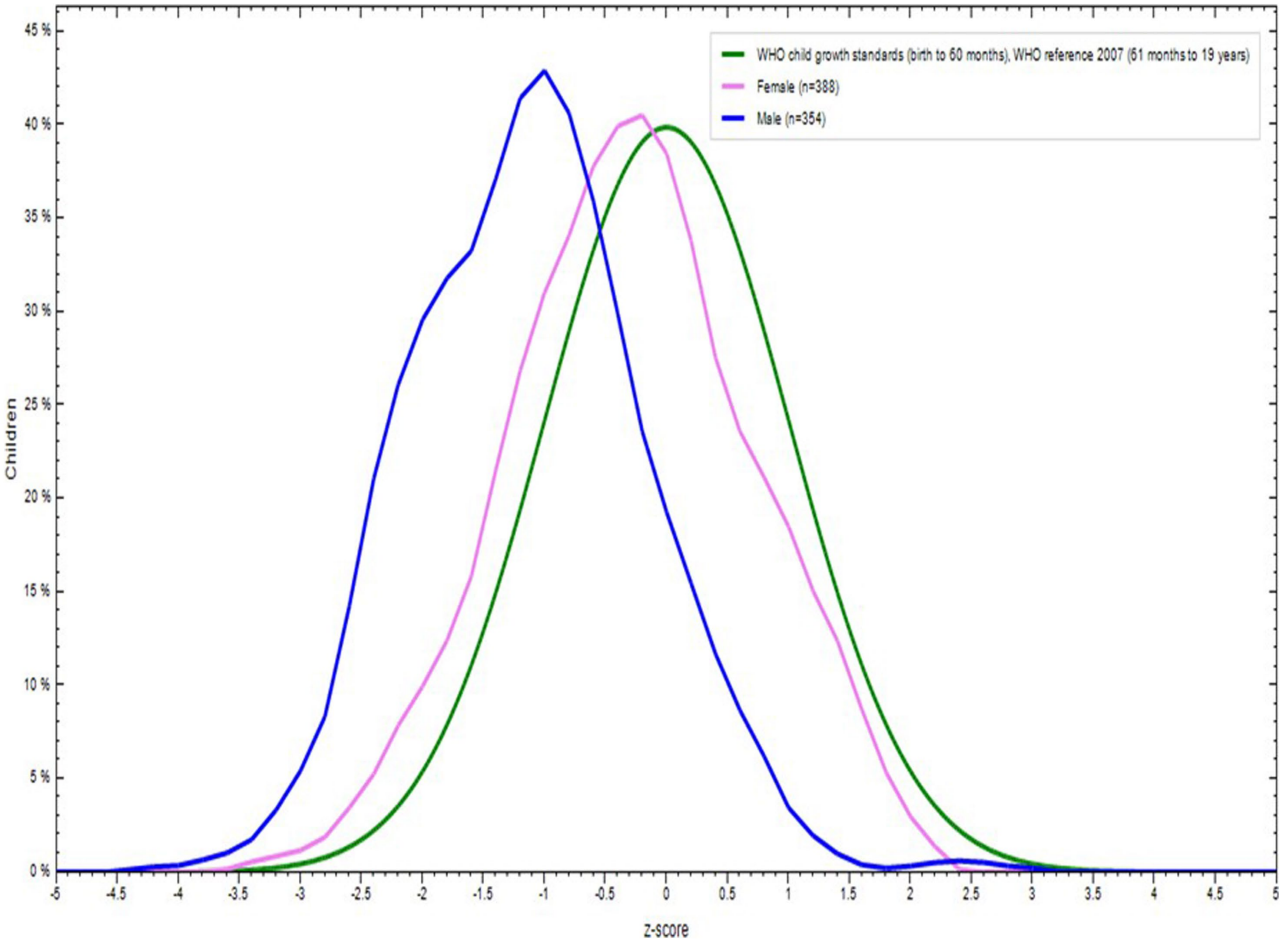
Body mass index (BMI) for Age Z score (BAZ) by sexes among school adolescents in Debre Berhan City, Ethiopia.

**Figure 4:**
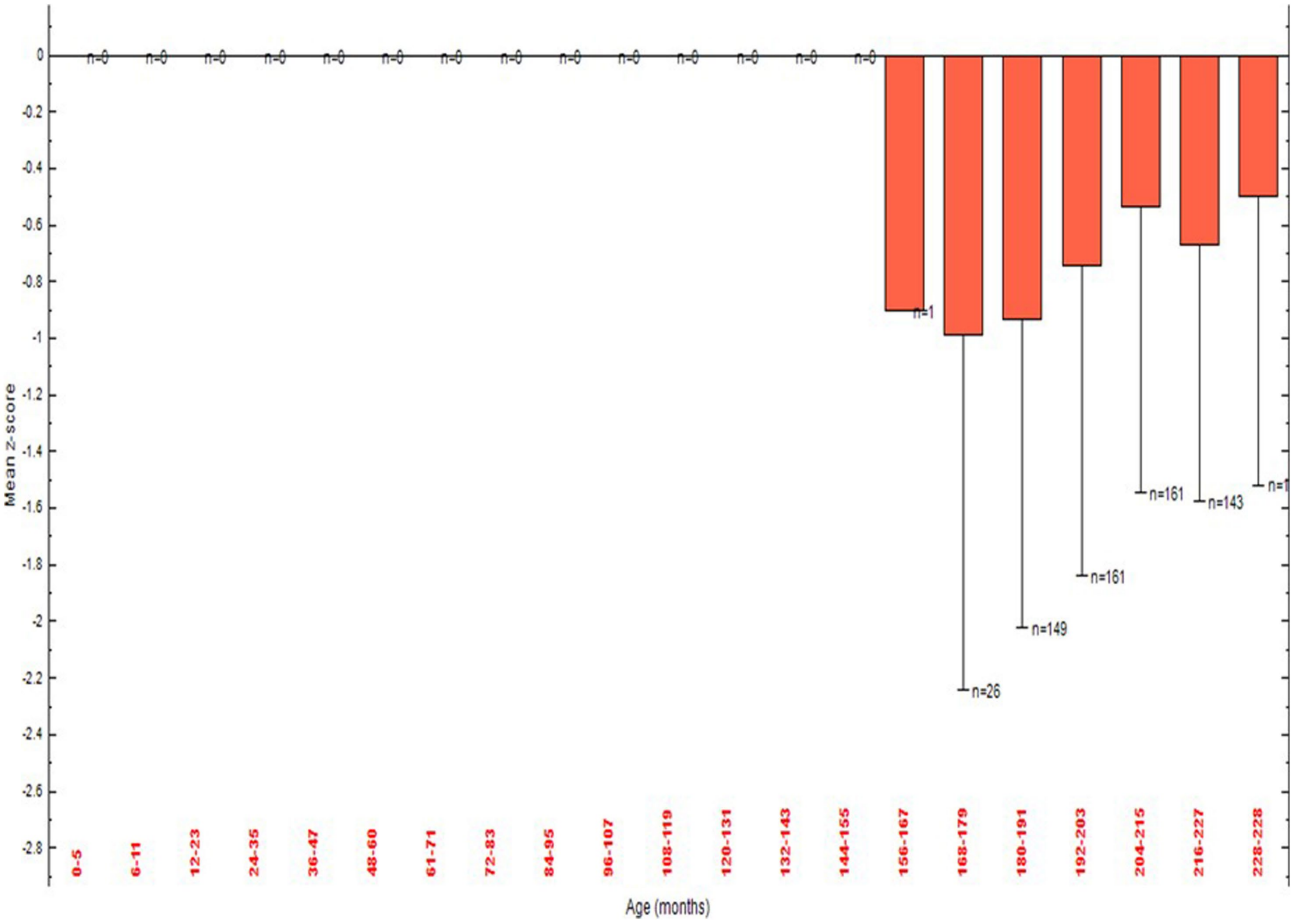
Body mass index (BMI) for Age Z score (BAZ) by age among school adolescents in Debre Berhan City, Ethiopia.

The percentage of all adolescents with Z-score was displayed in Figure 2. The percentage of adolescents with Z-score by sex of the school adolescents for both male (*n* = 354) and female (n = 388) was displayed in Figure 3. The mean Z-score of respondents with age in months of adolescents was displayed in Figure 4. The highest numbers of respondents by months were found in the age group 192–203 (*n* = 161) and 204–215 (*n* = 161).

**Figure 5:**
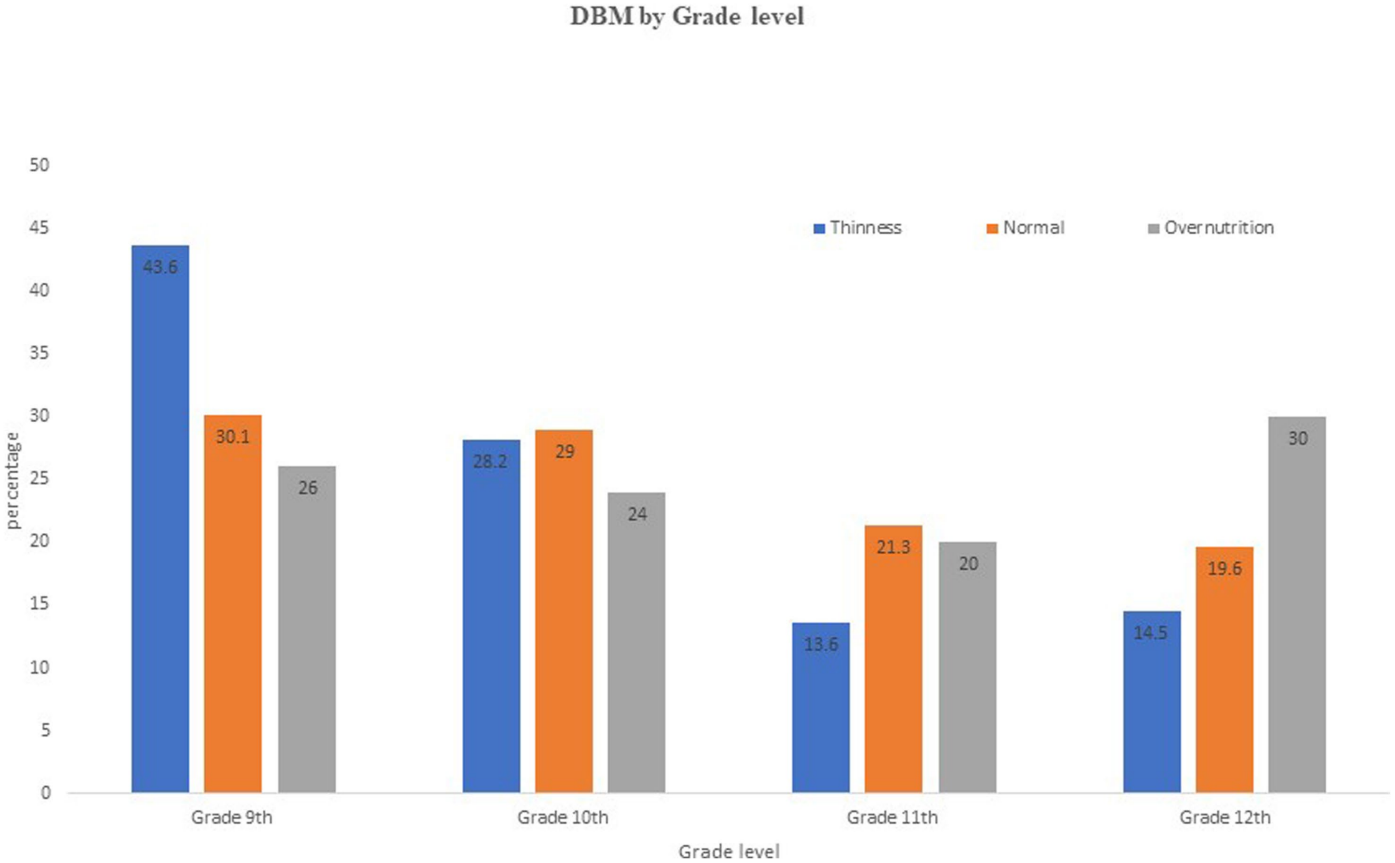
The DBM status of school adolescents by grade level in Debre Berhan City, Ethiopia.

The status of DBM was compared using the level of grade of the school adolescents. A high prevalence of undernutrition was observed in grade 9th students whereas a high level of overnutrition was observed among grade 12th students (Figure 5).

### 3.4. Factors associated with the double burden of malnutrition

In the multivariable multinomial logistic regression analysis model, factors such as age, sex, school type, minimum dietary diversity score, frequency of meals, home gardening practice, history of illness in the last 2 weeks, and knowledge of nutrition were the significant and independent predictors of DBM (either thinness or overweight/obesity) in-school adolescents (Table 3).

**Table 3:** Modeling the bivariable and multivariable multinomial logistic regression^1^ analysis model for factors associated with DBM among school adolescents in Debre Berhan City, Ethiopia, 2023 (*n* = 742).

Variables	Double burden of malnutrition (DBM)^$^			
	Thinness		Overweight/obesity	
	COR (95% CI)	AOR (95% CI)	COR (95% CI)	AOR (95% CI)
Age	0.77 (0.66, 0.89)^**^	**0.79 (0.67, 0.93)** ^**^	1.11 (0.90, 1.36)	1.27 (0.99, 1.63)
**Sex**				
Male	3.65 (2.29, 5.81)^*^	**3.86 (2.35, 6.32)** ^**^	0.13 (0.05, 0.340)^*^	**0.12 (0.05, 0.33)** ^**^
Female	1.00	1.00	1.00	1.00
**Type of school**				
Private	1.55 (1.02, 2.38)^*^	**2.24 (1.33, 3.77)** ^**^	1.74 (0.96, 3.16)	**5.03 (2.30, 10.99)**
Gov’t	1.00	1.00	1.00	1.00
**Dietary diversity score**				
Adequate	1.00	1.00	1.00	1.00
Inadequate	2.51 (1.49, 4.18)^**^	**2.29 (1.27, 4.14)** ^**^	5.01 (1.96, 12.81)^**^	**5.26 (1.88, 14.67)** ^**^
**Frequency of meal**				
<3	2.62 (1.54, 4.45)^**^	**2.09 (1.13, 3.89)**	7.38 (3.96, 13.76)^**^	**4.58 (2.15, 9.76)** ^**^
≥3	1.00	1.00	1.00	1.00
**Home garden practice**				
Yes	1.00	1.00	1.00	1.00
No	2.83 (1.87, 4.28)^**^	**2.31 (1.44, 3.67)** ^**^	5.19 (2.79, 9.66)^**^	**3.96 (1.92, 8.21)** ^**^
**History of illness**				
Yes	1.00	1.00	1.00	1.00
No	0.57 (0.37, 0.88)^*^	**0.57 (0.36, 0.93)** ^**^	0.53 (0.28, 0.98)^*^	0.61 (0.29, 1.26)
**Knowledge of nutrition**				
Good	1.00	1.00	1.00	1.00
Poor	3.39 (1.94, 5.92)^**^	**2.58 (1.41, 4.74)** ^**^	6.64 (2.36, 18.69)^**^	**4.96 (1.61, 15.33)** ^**^

^$^Normal is the reference group; ^*^P-value < 0.005; ^**^P-value < 0.001; AOR, Adjusted odds ratio; COR, Crude odds ratio; CI, Confidence interval; 1.00, reference category; Gov’t, government; ^1^Hosmer and Lemeshow’s test (*p*-value = 0.999). Bold values indicates the significant associated factors to give in focus.

The age of adolescents had a significant association with thinness compared to adolescents who had normal nutritional status. Every one-year increase in the age of school adolescents, the odds of thinness was increased by 0.79-point considering other factors constant [AOR = 0.79, 95% CL: (0.67, 0.93)] (Table 3).

Sex of school adolescents had a noteworthy association with thinness and overweight/obesity compared to the normal category. Male adolescents had 3.86 times [AOR = 3.86, 95% CL: (2.35, 6.32)] more likely to have thinness compared to female adolescents. Similarly, for adolescents who are in the overweight/obesity than normal category, male adolescents had 88% less likely [AOR = 0.12, 95% CL: (0.05, 0.33)] to have overweight/obese compared to female adolescents (Table 3).

School type had a significant association with thinness and overweight/obesity compared to the normal category. Private school adolescents were 2.24 times [AOR = 2.24, 95% CL: (1.33, 3.77)] more likely to have a significant association with thinness compared to government school adolescents. In the same way, private school adolescents were 5.03 times [AOR 5.03, 95% CL: (2.30, 10.99)] more likely to have a significant association with overweight/obesity compared to government school adolescents (Table 3).

The home gardening practice had a significant association with thinness and overweight/obesity compared to the normal category. School adolescents who had no home gardening practice were 2.31 times [AOR = 2.31, 95% CL: (1.44, 3.67)] more likely to have thinness compared to adolescents who had home gardening practice. Likewise, school adolescents who had no home gardening practice were 3.96 times [AOR = 3.96, 95% CL: (1.92, 8.21)] more likely to have overweight/ obesity compared to adolescents who had home gardening practice in their garden (Table 3).

The minimum dietary diversity score of adolescents had a significant association with thinness and overweight/obesity compared to the normal category. Adolescents who had inadequate minimum dietary diversity score were 2.29 times [AOR = 2.29, 95% CL: (1.27, 4.14)] more likely to have thinness compared to adolescents who had adequate minimum dietary diversity score. Correspondingly, adolescents who had inadequate minimum dietary diversity score were 5.26 times [AOR = 5.26, 95% CL: (1.88, 14.67)] more likely to have overweight/obesity compared to adolescents who had adequate minimum dietary diversity score (Table 3).

The frequency of meals had a significant association with thinness and overweight/obesity compared to the normal category. School adolescents who had less than three frequencies of meals were 2.09 times [AOR = 2.09, 95% CL: (1.13, 3.89)] more likely to have a significant association with thinness compared to adolescents who had three and greater than three frequencies of meals. Similarly, school adolescents who had less than three frequencies of meals were 4.58 times [AOR = 4.58, 95% CL: (2.15, 9.76)] more likely to have overweight/obese compared to adolescents who had three and greater than three frequencies of meals (Table 3).

History of illness in the last month had a significant association with thinness compared to the normal category. School adolescents who had no history of illness in the last month were 43% [AOR = 0.57, 95% CL: (0.36, 0.93)] less likely to have a significant association with thinness compared to school adolescents who had a history of illness in the last month (Table 3).

Knowledge of nutrition among adolescents had a significant association with thinness and overweight/obesity compared to the normal category. School adolescents who had poor knowledge of nutrition were 2.58 times [AOR = 2.58, 95% CL: (1.41, 4.74)] more likely to have thinness compared to school adolescents who had good knowledge of nutrition. Correspondingly, School adolescents who had poor knowledge of nutrition were 4.96 times [AOR = 4.96, 95% CL: (1.61, 15.33)] more likely to have overweight/obese compared to school adolescents who had good knowledge of nutrition (Table 3).

## 4. Discussion

The prevalence and predictors of DBM were examined in this study. In the research area, the overall prevalence of DBM was 21.5%. More particular, thinness 14.8% (95% CL: 12.3, 17.6%) and overweight/obesity 6.7% (95% CL: 5.0, 8.8%) were detected in school-aged adolescents. The significant and independent determinants of DBM in school-aged teenagers included age, sex, school type, minimum dietary variety score, frequency of meals, home gardening practice, history of illness in the previous months, and nutritional knowledge.

The overall prevalence of DBM in this study was higher than the previous studies conducted in Dessie town where the percentage was 14.5% (underweight 6.3% and overweight/obese 8.2%) (25), in Jimma Zone where the percentage was 18.7% (11.6% thinness and 7.1% overweight/ obesity) (32), in Southern Ethiopia where the percentage was 9.7% (4.7% thinness and 5% overweight/obesity) (30), in Addis Ababa where the percentage was 14.7% (6.2% underweight and 8.5% overweight/obesity) (31) and in Greece 22.5% (12.4% overweight/obese and 10.1 underweight) (20). However, it was lower than studies done in southwest Ethiopia where the percentage was 33.2% (28% underweight and 5.2% overweigh/obese) (34), in Gondar city where the percentage was 40.1% (undernourished 12.9%, overweight 21.3% and obese 5.9%) (26), in Bahir Dar city where the percentage was 28.1% (underweight 15%, overweight 8.4%, and obesity 4.7%) (28), in Finote Selam Town where the percentage was 48.9% (underweight 46.2% and overweight 2.7%) (40), in Indonesia where the percentage was 27% (16% thinness and 11% overweight/obese) (21), in India where the percentage was 55.6% (47% underweight, 5.9% overweight, and 2.7% obese) (23), in south India where the percentage was 39.2% (14.7% severely thin, 15.8% thin, 6.9% overweight and 2.2% obese) (22), in southern Ethiopia where the percentage was 30.9% (underweight 19.7% and overweigh/obesity 11.2%) (27), and in Adama city where the percentage was 25.6% (21.3% underweight, 3.3% overweight, and 1.0% obese) (24). This mismatch may be brought on by differences in sociodemographic characteristics such as family educational and occupational diversity, access to information on nutrition and feeding practice, and nutrition knowledge of adolescent. Furthermore, for this perceived variation, other factors like outcome classification methods (since this study classify the outcome into three classifications), sample size, and analysis methodology may be taken into account.

Compared to school adolescents with normal nutritional status, age demonstrated a significant association with thinness. When all conditions are held constant, there is an approximately 0.79-point rise in the odds of thinness for every one-year increase in age. As age increases, the probability of getting thin increases. This finding is similar to the study conducted previously (28, 43). This may be related to the fact that some adolescents think that our shape will be disfigured and disturbed when we take much amount of food (44, 45). Due to this assumption, they will reduce food intake and exposed to thinness.

Sex among school-aged adolescents was significantly associated with both thinness and overweight or obesity, in contrast to the normal group. Male adolescents were approximately four times as likely to be thin as compared to female teenagers. This result was in line with earlier research (23, 25, 30, 40). On the other hand, male adolescents had an 88% lower likelihood of being overweight or obese. This outcome is consistent with the available data (46–48). The probable reason may be that, in contrast to females, males lead an active lifestyle that involves energy-demanding activities. Moreover, since girls tend to stay at home, they might have better access to food than males do. This might make males more prone to being thin.

School type had a substantial connection with thinness and overweight/obesity compared to the normal category. Equated to government school adolescents, adolescents in private schools were more than two times more likely to have a significant connection with thinness. This result was not supported by the previous studies (30, 31). This dissimilarity may due to variation in a model of analysis since this study uses a multinomial regression analysis model instead of a binary logistic regression analysis model. In a similar vein, private adolescents were more than five times more likely to have a significant association with overweight/obesity than those in government schools. This finding was consistent with the previous studies (30, 31, 47, 49–51). The greater financial access of student households may be the possible reason. Additionally, they might receive specific assistance and special care from their family, such as access to transportation.

Compared to the normal category, the odd of home gardening demonstrated a strong correlation with thinness and overweight/obesity. School teenagers who did not engage in home gardening had nearly two and half times higher likelihood of becoming thin than those who did. This finding is similar to a study conducted in Rwanda (52). Participants in home gardening have much higher results for child nutrition and nutrition security than non-participating households. Likewise, school-aged children and adolescents who did not engage in home gardening were nearly four times more likely to be overweight or obese than those who did home gardening practice. This result is similar from the previous studies. Home gardening had a positive impact on anthropometric measures and more generally on children’s health (53, 54). This indicates that the access to many nutrients can be increased by growing our own produce at home, including fruits, vegetables, and other significant plant sources. Teenagers may be exposed to both sides of malnutrition in the absence of such situations. This suggests that not engage in home gardening practice can acts as the driver factor of both side of malnutrition.

Adolescents’ minimum dietary diversity scores significantly predicted thinness and overweight or obesity as compared to the normal group. Adolescents with inadequate minimum dietary variety scores had nearly two and half times greater likelihood of being thinner than those with adequate minimum dietary diversity scores. This result was in line with earlier research (29, 43, 55, 56). Respondents who had adequate minimum dietary diversity scores had a high probability to be prevented from developing thinness. Also, adolescents with inadequate minimum dietary variety scores were more than five times more likely to be overweight or obese than those with adequate minimum dietary diversity scores. Higher dietary diversity had a strong association with reductions in overweight or obese according to the research done in the former time (56–59). This could be as a result of the association between dietary diversification and decrease in the total calories from carbohydrate, fat, saturated fat, and high lipodensity cholesterol. This will result in a reduction in the percentage of the food groups that are consumed and is linked to little weight gain. This shows that both sides of malnutrition may be caused by poor dietary diversity scores.

Compared to the normal group, frequency of meals had a significant correlation with the odds of thinness and overweight/obesity. When compared to school teenagers who ate three or more times per day, those who ate less frequently had a more than two times larger likelihood of having a meaningful link with thinness. This finding was consistent with the study conducted in different parts of Ethiopia (25, 30, 34). Corresponding to this, adolescents who ate less frequently were nearly five times more likely to have a meaningful link with overweight/obesity than adolescents who had three and larger frequency of meals. This finding is consistent with the existing studies (60–62). According to these studies, eating out less than three meals frequently had a significantly positive effect on overweight and obesity. Eating timely and taking adequate amounts of food may decrease excessive eating at one time and avoid over-accumulation of fat in the body. Furthermore, low meal frequency is also associated with inadequate nutritional intake in terms of quantity, variety, and quality. Because adolescent growth is the fastest and they have greater nutritional needs at this time, eating infrequently will result in underweight or thinness. On the other side, people run the risk of becoming overweight or obese if they follow fewer frequency each day but consume more of foods at a time. This indicates that both sides of malnutrition may be caused by low meal frequency.

When compared to the normal group, the odd of thinness was significantly correlated with no illness history during the previous month. When compared to school adolescents who had a history of illness in the previous month, those with no history of illness were 43% less likely to have an association with thinness. According to the findings of this study per the available information, respondents who had a history of illness in the past were exposed to thinness (30, 34). This may be connected to the idea that being ill will reduce intake and exposure to certain diseases.

Teenagers’ nutritional knowledge was significantly correlated with thinness and overweight/obesity when compared to the normal category. When compared to school adolescents with strong good nutrition awareness, those with poor nutrition knowledge were more than two and half times more likely to be skinny. The previous studies reported that knowledge of nutrition improves the nutritional status of respondents (63–65). Corresponding to this, school teenagers with poor nutrition knowledge were nearly five times more likely to be overweight or obese than those with strong nutrition knowledge. In the same way, the knowledge of nutrition decreases the overweight and obesity of respondents in the preceding studies (66, 67). The likelihood of preventing both undernutrition and overnutrition will rise as our knowledge of nutrition grows. This shows that poor understanding nutrition may be a driving force for both sides of malnutrition.

In a nutshell, the results of this study have advanced support for the goals of the national food and nutrition strategy, which aims to increase the intake and use of a varied and nutritious diet to guarantee adolescents optimal health over the course of their lives in the nation. Its primary goal is to lessen the prevalence of DBM across all population groups, including school-aged adolescents nationally. Lastly, the implementation of double-duty interventions will have a double impact on DBM prevention.

Regarding the strength and limitations of the study, it has the strength of using an advanced model of analysis, which is using a multinomial regression model of analysis compared to the dominant binary logistic regression model in the existing body of research. On the other hand, this study was limited by recall bias and reporting for some of the variables like dietary practice and history of illness. However, these limitations were minimized by detail probing and remembering the issues using different techniques such as introducing what were pertinent events when they experienced such issues.

## 5. Conclusion

The status and DBM predictors were examined in this study. The overall prevalence of DBM was 21.5% (14.8% thinness and 6.7% overweight/obesity). This prevalence is higher compared with the national and regional prevalence that found to be a public health concern. The significant and independent determinants of DBM in school-aged teenagers were age, sex, school type, minimum dietary variety score, frequency of meals, home gardening practice, history of illness in the previous 2 weeks, and nutritional awareness. Thus, the federal minister of health, particularly the Debre Berhan City health office in collaboration with education office should design double duty interventions that aimed at reducing the two sides of DBM which comprise both thinness and overweigh/ obesity among adolescents. Especially, the intervention packages like double-duty interventions should focus on how to improve minimum dietary variety score, follow appropriate frequency of meals, grow home garden vegetables and fruits, get treatment on illness, and increase knowledge of nutrition.

## Data availability statement

The data used to support the findings of this study are available from the corresponding author upon reasonable request.

## Ethics statement

The guidelines of the Helsinki Declaration and good clinical practice (GCP) were followed in the conduct of the study (68). The Jimma University Institutional Review Board (IRB) accepted the research protocol and ethical approval was obtained. The study objectives were communicated to Debre Berhan city administrators, the zonal education office, kebele administrators, and other pertinent authorities through a letter produced by the Jimma University IRB office in order to improve and facilitate support and cooperation. All data collection procedures followed the rules and regulations of the Ethiopian National Research Ethics and Jimma University Research Ethics. Written informed consent to participate in this study was provided by the participants’ legal guardian/next of kin.

## Author contributions

LG, BA, and TB agreed on the journal to which the article was submitted, gave final approval of the version to be published, contributed to data analysis, drafting, or revising the paper, and agreed to be responsible for all parts of the work. All authors contributed to the article and approved the submitted version.

## Funding

This work was supported by Jimma University in collaboration with Debre Berhan University. However, the universities have no role in the design of the study, collection, analysis, and interpretation of the data and in writing the manuscript.

## Conflict of interest

The authors declare that the research was conducted in the absence of any commercial or financial relationships that could be construed as a potential conflict of interest.

## Publisher’s note

All claims expressed in this article are solely those of the authors and do not necessarily represent those of their affiliated organizations, or those of the publisher, the editors and the reviewers. Any product that may be evaluated in this article, or claim that may be made by its manufacturer, is not guaranteed or endorsed by the publisher.
